# Renal outcome in adults with renal insufficiency and irregular asymmetric kidneys

**DOI:** 10.1186/1471-2369-5-12

**Published:** 2004-10-05

**Authors:** Guy H Neild, Gill Thomson, Dorothea Nitsch, Robin G Woolfson, John O Connolly, Christopher RJ Woodhouse

**Affiliations:** 1 Institute of Urology and Nephrology, University College London, W1T 3AA, UK; 2 Renal Unit, Middlesex Hospital (UCL Hospitals), Mortimer St., London, W1T 3AA, UK; 3 Department of Epidemiology and Population Health, London School of Hygiene and Tropical Medicine, Keppel St., London, WC1E 7HT, UK

## Abstract

**Background:**

The commonest cause of end-stage renal failure (ESRF) in children and young adults is congenital malformation of the kidney and urinary tract. In this retrospective review, we examine whether progression to ESRF can be predicted and whether treatment with angiotensin converting enzyme inhibitors (ACEI) can delay or prevent this.

**Methods:**

We reviewed 78 patients with asymmetric irregular kidneys as a consequence of either primary vesico-ureteric reflux or renal dysplasia (Group 1, n = 44), or abnormal bladder function (Group 2, n = 34). Patients (median age 24 years) had an estimated GFR (eGFR) < 60 ml/min/1.73 m^2 ^with at least 5 years of follow up (median 143 months). 48 patients received ACEI. We explored potential prognostic factors that affect the time to ESRF using Cox-regression analyses.

**Results:**

At start, mean (SE) creatinine was 189 (8) μmol/l, mean eGFR 41 (1) ml/min 1.73 m^2^, mean proteinuria 144 (14) mg/mmol creatinine (1.7 g/24 hrs). Of 78 patients, 36 (46%) developed ESRF, but none of 19 with proteinuria less than 50 mg/mmol and only two of 18 patients with eGFR above 50 ml/min did so. Renal outcome between Groups 1 and 2 appeared similar with no evidence for a difference. A benefit in favour of treatment with ACEI was observed above an eGFR of 40 ml/min (p = 0.024).

**Conclusion:**

The similar outcome of the two groups supports the nephrological nature of progressive renal failure in young men born with abnormal bladders. There is a watershed GFR of 40–50 ml/min at which ACEI treatment can be successful at improving renal outcome.

## Background

Nearly half the children and young adults who develop end-stage renal failure (ESRF) have asymmetric irregularly shaped kidneys [1]. This appearance, often referred to as bilateral renal scarring, is frequently associated with vesico-ureteric reflux (VUR) and sometimes with a history of urinary tract infection (UTI). It is generally a consequence of congenital malformations of the kidneys and urinary tract and is variously described as `reflux nephropathy' or `chronic pyelonephritis.'

Such patients fall into two broad groups. Firstly, there is a group who appear to have normal bladders without outflow obstruction and normal calibre ureters when not micturating, described as having either primary VUR or primary renal dysplasia. Secondly, there is a group with some form of bladder outflow dysfunction which causes a secondary VUR and dilated upper urinary tracts, of which a posterior urethral valve (PUV) in males is the most common cause.

The primary group have a bimodal presentation. Commonly they present in childhood with UTI; the rest present in early adult life with renal insufficiency and often with no preceding history of UTI [2-6]. Traditionally the diagnosis was made by recognising the characteristic appearance of calyceal clubbing and irregular `scarring' of the kidney on intravenous urography (IVU) [7,8]. With significant renal insufficiency, however, these changes can be impossible to see clearly by IVU [2], and the irregular, asymmetrical kidney is more sensitively visualised by ^99 m^Tc-dimercaptosuccinic acid (DMSA) renography [9,10]. In this adult population a micturating cysto-urethrogram (MCU) frequently will not show evidence of VUR as reflux usually ceases spontaneously in childhood [2,4,5]. In fact, the finding of VUR is a weak predictor of renal damage in children admitted with an UTI [11].

The appearance of proteinuria and *progressive *renal failure indicates glomerular capillary hypertension (glomerular hyperfiltration) and progressive focal and segmental glomerulosclerosis (FSGS) [12,13]. Risk factors for patients with reflux nephropathy developing progressive renal failure after childhood are proteinuria, renal insufficiency, bilateral scarring of the kidneys and hypertension [2,4,5]. Patients with congenital bladder outflow obstruction and secondary reflux, however, have usually been excluded from such outcome studies, and very little has been published from a nephrological perspective about their long-term outcome.

In this retrospective observational review, from a large, single centre nephro-urological practice, we have examined the natural history and progression to ESRF of patients with primary and secondary reflux with asymmetric irregular kidneys and moderate to severe renal insufficiency. We have tested the null hypothesis of no difference in renal outcome between patients with primary and secondary reflux.

## Methods

### Patients

Patients with bilaterally scarred kidneys and glomerular filtration rate (GFR) 15–60 mls/min/1.73 m^2 ^were identified from a review of the records of outpatients and of patients receiving renal replacement therapy at the Renal Unit of the Middlesex Hospital (UCL Hospitals Trust). Most patients had been referred, as adolescents, from the nephrology and urology clinics at the Great Ormond Street Hospital for Children. All patients had renal scarring confirmed by DMSA or ^99 m^Tc-mercaptoacetyltriglycine (MAG-3) renography, although most patients had undergone extensive investigations.

For inclusion in this study, patients had:

• an isotopic ^51^Cr-edetic acid (EDTA) GFR < 60 ml/min/1.73 m^2^; or estimated GFR < 60 ml/min/1.73 m^2^

• apparently stopped growing and with a steady body weight (so that plasma creatinine could be used to estimate serial GFRs), and

• data for at least 5 years of follow up.

Patients specifically excluded from this study were those with bladder exstrophy, neuropathic bladders, or any form of urinary diversion (conduit or reservoir). In our analysis, the patients were divided into two broad groups:

Group 1: those with normal calibre ureters and normal bladders (Primary group)

Group 2: those with megaureters, hydronephrosis and abnormal bladders (Secondary group).

### Data

*Glomerular filtration rate *(GFR) was estimated by single exponential analysis of the plasma clearance of ^51^Cr-edetic acid (EDTA) following a single intravenous injection with blood samples taken after 2 and 4 hours [14]. Plasma (PCr) and urine creatinine concentration were measured by the Jaffe technique using an autoanalyser (Chemlab Instruments, Hornchurch, UK) and urinary protein (Uprot) by turbidometric assay following precipitation with trichloroacetic acid.

GFR was estimated by different formulae and compared with the isotopic GFR. The Jelliffe formulae (I and II) [15,16] have been shown to approximate most closely low values of GFR when compared with the inulin clearance [17].

Jelliffe I (ml/min/1.73 m^2^) [15]:- "(100 × 88/ PCr μmol/l) - 12" for males; and "(80 × 88/creatinine μmol/l) - 7" for females.

Jelliffe II (ml/min/1.73 m^2^) [16]:- "(98–0.8(age-20)) × 88/ PCr μmol/l" for males. (Jelliffe II × 0.9 for females). [The original formulae used creatinine mg/dl. Conversion to μmol/l introduces the factor 88].

We found that a mean of these 2 formulae gave closer approximations to measured isotopic GFR than the other methods. We have termed this mean value `estimated GFR' (eGFR). All measured values of ^51^Cr EDTA GFRs were compared with the eGFR calculated using the contemporary value of plasma creatinine. 151 values of corrected isotopic GFR (ml/min/1.73 m^2^) from a range of 12–60 ml/min/1.73 m^2 ^were found to have no significant bias (-0.34 ml/min/1.73 m^2 ^with a 95% CI of -1.19 to 0.51 ml/min/ 1.73 m^2^) and an agreement within limits of -11.0 to 10.3 ml/min/1.73 m^2^.

*Normal bladder: *patients presenting after adolescence were considered to have a normal bladder if they had no bladder outflow symptoms, a normal urine flow rate (> 15 ml/sec) and no residual urine volume seen by ultrasound after voiding.

*Declining renal function: *the rate of progression of CRF, -delta GFR (`-ΔGFR'), was calculated as the rate of change of eGFR and is shown as ml/min/year.

*Proteinuria: *was initially measured as the amount of protein (g) in a 24-hour urine collection. Since 1995 proteinuria was more commonly measured on a random (spot) sample of urine at clinic visits with the proteinuria expressed as mg protein/mmol creatinine (normal laboratory range 0–13 mg/mmol). As all 24 hour urine data (Uprot) included creatinine excretion (mmol/24 hours) we have been able to calculate protein/creatinine ratios (Up/Cr). Using the data from 161 separate 24-hour collections, we assessed how proteinuria in g/day predicts protein/creatinine ratios. Paired values ranged from 0.1–8.6 g/day and 8–700 mg protein/mmol creatinine, with a high correlation (r = 0.90). The regression equation was [Up/Cr = 90.3 × Uprot^0.94^].

*Hypertension *was defined as either blood pressure consistently > 140/85 mmHg, or patients receiving blood pressure lowering therapy.

*End-stage renal failure *(ESRF): was taken to be the date when the patient began dialysis. To calculate changes in renal function with time the eGFR was assumed to be 8 ml/min at this time.

*Outcome: *Renal outcome was defined as having reached ESRF or not at last review.

### ACEI therapy

Since June 1986 some patients were started on ACEI therapy when anti-hypertensive therapy was required. In addition, some anti-hypertensive regimens were changed to ACEI therapy. A small group with blood pressure <140/85 were started on ACEI therapy because of increasing proteinuria. Some patients never received ACEI because ESRF was reached before the use of ACEIs in renal insufficiency had become routine.

Patients, who were started on ACEI for hypertension, were advised to restrict their salt intake and the initial aim was for a blood pressure of ≤ 130/70. If ACEI alone did not lower the blood pressure to the target a diuretic was added. The latter was not always possible in Group 2 patients who might already have features of hypovolaemia secondary to their renal tubular pathology. No patient ever received any immunosuppressive drug.

### Data collection

Only data from the start and end of the study are presented from all 78 patients. Of the 48 patients who were treated with ACEI, we also report data on 28 of the patients (for whom it was available for at least 18 months before and 48 months after the introduction of ACEI.) at the start of ACEI therapy and again at 2 years after start of therapy.

### `Statistical Analysis'

Means were compared within groups by paired samples t-test, and between groups by Wilcoxon rank-sum test. The time scale was time since birth, since the setting was of a congenital disease eventually leading to renal failure. It was checked graphically that there was no obvious pattern to loss to follow up over time. Renal outcome (reaching ESRF or not) was compared between groups using Kaplan-Meier survival plots. Outcomes were quantified as the median survival outcome in months (with 95% Confidence Intervals [CI]). This is equivalent to the median time for 50% of the group to reach ESRF. Differences in renal outcome over time were tested using log-rank tests. For graphical examination of the proportionality, continuous variables were grouped into approximate tertiles. The Cox proportional hazards model was chosen for further analysis. Continuous variables were centred on the mean. Protein/urine creatinine ratios were log-transformed for further analyses. Treatment with ACEI was entered as a time-changing variable to take account of the variable start of treatment after referral. Univariable regression models estimated crude (unadjusted) effects of the prognostic variables. Since it is possible that the effect ACEI depends on the GFR at start of treatment (entered as continuous variable), especially in patients with moderate to severe renal insufficiency, an interaction between those variables was fitted. Variables were entered in the multivariable model in a stepwise forward fashion. Analyses were performed using SPSS for Windows v10.1. and Stata 8 (Stata Corporation, Texas, USA).

## Results

### Demography

Data from 78 patients, who were first seen between December 1969 and February 1988, were analysed. Demographic details are presented in Table 1. The age of patients at the start of the study period ranged from 15–49 years (median 23 years) with one lady aged 65 years.

**Table 1:** Demographic Details At Entry (n = 78). Data shown are means (SE); Dates (month/year) are medians. Because isotopic GFRs were not always performed this data is not shown in Table, but 26 Group 1 patients had a mean (SE) isotopic-GFR of 41.2 (2.1) ml/min/1.73 m^2 ^with a contemporaneous mean eGFR of 44.2 (2.3) ml/min/1.73 m^2^, and the 26 Group 2 patients with an isotopic GFR of 40.2 (2.3) had an eGFR 41.5 (2.0) ml/min 1.73 m^2^.

	***Group 1 (Primary reflux)***	***Group 2 (Secondary reflux)***	***TOTAL***
Total	44	34	78
Male:Female	22:22	32:2	54:24
Date at start (median)	8/1988	6/1986	2/1987
Age (year)	28.9 (1.6)	22.2 (1.4)	26 (1.1)
Creatinine (μmol/l)	178 (9.0)	203 (13)	189 (7.7)
eGFR (ml/min)	42.3 (1.8)	40.3 (2.1)	41.4 (1.3)
Proteinuria (g/d)	1.63 (0.19)	1.83 (0.33)	1.72 (0.2)
Protein/creatinine (mg /mmol)	136 (14)	154 (27)	144 (14)
Hypertension	18 (41%)	5 (15%)	23 (29%)
Treatment with ACEI	32 (73%)	16 (47%)	48 (62%)
Treatment date (median)	4/1992	8/1993	11/1992
**Total months of follow up**	145 (11)	143 (9)	144 (7)

Group 1 (n = 44; 50% female) were patients who had primary VUR or primary renal dysplasia. Twenty two patients (71% female) presented in childhood with a UTI. In each case, MCU performed at a median age of 7 years showed reflux which was either bilateral (82%) or unilateral (18%). In contrast, the remainder (n = 22) presented at a median age of 24 years. Only 32% were female and they almost invariably presented either with hypertension after starting a contraceptive pill or with complications during pregnancy. Only 2 had had a MCU performed and neither showed VUR.

Group 2 patients (n = 34; 6% female) had the following diagnoses: PUV (n = 15), prune belly syndrome (n = 2), single dysplastic kidney with megaureter (n = 2), renal dysplasia with abnormal bladder function (n = 2), bilateral megaureters (n = 1), megacystis and megaureters (n = 4), and finally a group (n = 8) in whom the initial diagnosis (pre-1979) had included "bladder neck obstruction". Six of these had megacystis and megaureters and might now be termed `pseudo-prune belly syndrome'.

### Renal outcome

*By Group: *The median survival time of Group 1 versus Group 2 was compared by log rank test: 231 months (95% CI: 153–309) vs. 162 months (95% CI: 135–189) respectively, p = 0.35. There was no evidence for a major difference in renal outcome between these patients with primary and secondary reflux. Thus for subsequent analyses the data from all 78 patients was combined.

**Figure 1:**
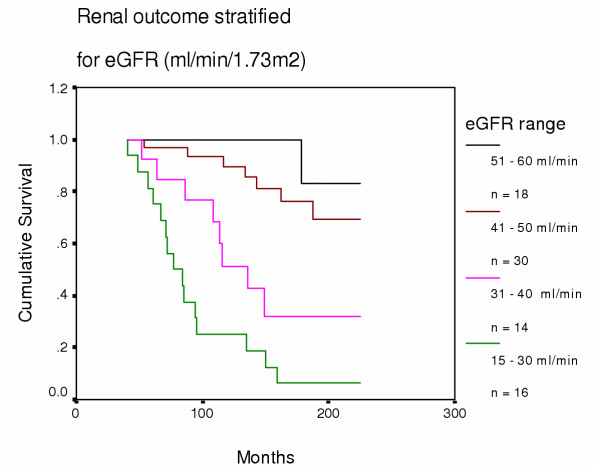
**Renal outcome stratified for eGFR (ml/min/1.73 m^2^) at start. **eGFR 51–60 vs 41–50: p = 0.17; eGFR 41–50 vs 31–40: p = 0.004; eGFR 31–40 vs 15–30: p = 0.041.

*By eGFR: *We compared renal outcome for all patients after they were stratified by initial eGFR into four groups (15–30, 31–40, 41–50, and 51–60 ml/min/1.73 m^2^) (see Fig 1). Of 18 patients with eGFRs >50 ml/min, only 2 (11%) reached ESRF: however, eGFR still declined in the other 16 by 1.30 ml/min/yr and mean proteinuria rose from 35 to 55 mg/mmol creatinine, despite 12 patients (75%) receiving ACEI.

**Figure 2:**
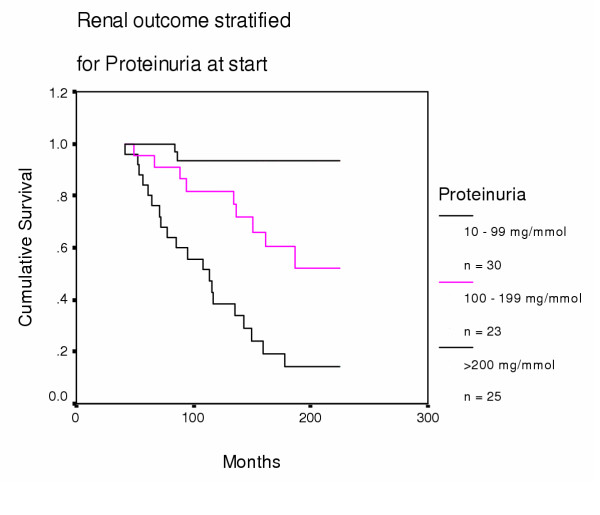
**Renal outcome stratified for proteinuria (mg/mmol) at start. **10 – 99 vs 100–199 mg/mmol: p = 0.009; 100–199 vs >200 mg/mmol: p = 0.002.

*By Proteinuria: *We compared renal outcome for all patients after they were stratified by initial proteinuria into three groups (0–99, 100–199, and ≥ 200 mg/mmol) (Fig 2). The great significance of proteinuria is emphasised further by the observation that seven patients with proteinuria ≥ 200 mg/mmol reached ESRF despite initial eGFRs ≥ 40 ml/min. In contrast, none of 19 patients with proteinuria <50 mg/mmol creatinine at start developed ESRF after a median follow up of 160 months (range 87–227).

Proteinuria increased with time and declining function in both Groups (Table 2) but levels were consistently higher in Group 2 compared with Group 1 patients (Table 1). There was a strong correlation between rate of loss of function and proteinuria at start (R = 0.63, p < 0.0001), and end of study: (R = 0.69).

**Table 2:** Creatinine, eGFR, proteinuria and ACE-I stratified by renal function at outset. Data are medians (range). Proteinuria*: b) vs c) p = 0.06, c) vs d) p = 0.031; **-Δ eGFR**^†^: b) vs c) p= 0.14, c) vs d) p = 0.012; **Total -ΔGFR **is the rate of change of function in ml/min/yr from start to last follow up; **Rx ACEI **is the percentage of patients receiving ACEI treatment

***Renal Function groups***	***N=***	***Creatinine***	***eGFR***	***Proteinuria***	***-Δ eGFR Total***	***-Δ eGFR post-ACEI***	***Rx ACEI***
		μmol/l	ml/min/1.73 m^2^	mg/mmol	ml/min/yr	ml/min/yr	

a) 15–30 ml/min	16	295 (220–450)	24	209 (57–680)	2.94 (0.55–5.7)	2.63 (1.51–4.3)	38%
b) 31–40 ml/min	14	198 (160–233)	36	200 (71–275)*	3.05 (0.95–7.4)	1.7 (0.9–3.45)	50%
c) 41–50 ml/min	30	160 (130–185)	46	100 (10–276)*	1.71 (0.44–8.21)^†^	1.68 (0.66–7.85)	77%
d) 51–60 ml/min	18	130 (115–153)	55	38* (10–250)	1.34 (0.24–3.41)^†^	1.76 (0.24–3.73)	72%

### ACEI treatment

48 patients commenced ACEI therapy at a median of 48 months (range 0–311) after the start of the study, by which time their median eGFR had fallen from 46 (range 15–60) to 36 (10–60) ml/min/1.73 m^2^.

### Effect on renal outcome

Table 3 shows the results of both the univariable and multivariable analyses. For every variable entered into the model the assumption of proportionality of hazards was met. It was notable, given the small number of patients of our sample, that we were able to detect an interaction between treatment with ACEI and renal function at treatment start. The effect of ACEI was estimated to have its main effects just above a GFR of 40 ml/min. In the crude, as well as in the full model, neither sex nor type of reflux seem to have a significant effect upon time to ESRF since referral. At entry to study, both the amount of proteinuria and eGFR were important prognostic variable towards ESRF in crude as well as adjusted analyses. There was one 65 year old lady. Refitting the final model omitting this record did not affect the estimates.

**Table 3:** Estimated crude and adjusted hazard ratios for incidence of ESRF in all patients. *full model includes all variables, since analyses were conducted on the age-scale, effects are taking account of current age; **interaction parameters (95%CI): crude model: -0.088 (-0.162,-0.014); p = 0.019 full model: -0.093 (-0.174,-0.012); p = 0.024; ^#^effect of 100 mg/mmol creatinine = (displayed hazard ratio)^0.7 ^effect of 200 mg/mmol creatinine = (displayed hazard ratio)^1.4;^^ ##^effect of 10 ml/min/1.73 m^2 ^decrease = (displayed hazard ratio)^2 ^effect of 15 ml/min/1.73 m^2 ^decrease = (displayed hazard ratio)^3^

		Hazard ratios					
		
Estimated effects of	categories/unit	crude	95%CI	p-value	Adjusted*	95%CI	p-value
Gender	female	1.00			1.00		
	male	1.17	(0.55, 2.50)	0.677	0.55	(0.21,1.39)	0.205
Type of reflux	primary	1.00			1.00		
	secondary	1.28	(0.65, 2.55)	0.478	1.73	(0.73, 4.06)	0.211
Proteinuria	per 50 mg/mmol proteinuria increase^#^	1.71	(1.33, 2.20)	<0.001	1.50	(1.17, 1.91)	0.001
eGFR**	per 5 ml/min/1.73 m^2 ^decrease^##^	1.38	(1.16, 1.64)	<0.001	1.29	(1.05, 1.58)	0.016
ACE inhibitor**	at 30 ml/min/1.73 m^2^	0.53	(0.22, 1.29)	0.162	0.67	(0.27, 1.67)	0.393
	at 35 ml/min/1.73 m^2^	0.34	(0.12, 1.00)	0.051	0.42	(0.14, 1.26)	0.121
	at 40 ml/min/1.73 m^2^	0.22	(0.06, 0.84)	0.027	0.27	(0.07, 1.04)	0.058
	at 45 ml/min/1.73 m^2^	0.14	(0.03, 0.73)	0.02	0.17	(0.03, 0.91)	0.039

### Effect on rate of progression and proteinuria

**Figure 3:**
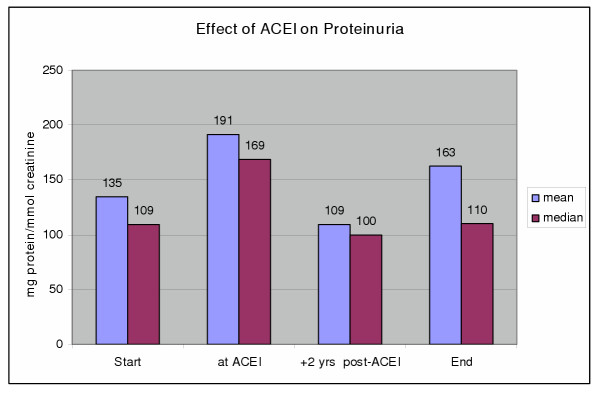
**Effect of ACEI on Proteinuria. **Time points are 1) start of study, 2) at begin of ACEI therapy, 3) 2 years after begin ACEI; 4) at end of study. Proteinuria* *at ACEI *vs *+2 years post-ACEI*; p < 0.0001.

We examined the effect of ACEI on 28 patients with deteriorating function for whom data was available for at least 18 months before and 48 months after the introduction of ACEI. ACEI reduced the rate of loss of renal function during the first 24 month period of follow-up, but the benefit was greater in the subsequent follow-up period with the rate slowing from a median of -1.86 before ACEI to -1.48 ml/min/yr (p = 0.007). ACEI treatment was associated with a reduction in proteinuria after 24 months of therapy. However, proteinuria had increased at last follow up owing to loss of the anti-proteinuric effect in half the group (Fig 3).

Analysis of 30 patients who did not receive an ACEI provides indirect support of benefit from this treatment. The median rate of loss of renal function for 25 of these patients who started with a eGFR ≤ 50 ml/min was 2.80 ml/min/yr and if initial proteinuria was ≥ 50 mg/mmol, (n = 23) the rate of loss of renal function was 3.0 ml/min/yr.

### Blood pressure

At the start of the study 18 (41%) Group 1 patients and 5 (15%) Group 2 patients were hypertensive. Group 2 patients tended to be normotensive and some were started on an ACEI for proteinuria. At ESRF or last follow up, all patients were on conventional anti-hypertensive therapy or ACEI, except for two Group 2 patients.

## Discussion

There is a consensus that patients with renal insufficiency *and *proteinuria have progressive renal failure, that the rate of decline of function is proportional to the magnitude of the proteinuria, and that angiotensin antagonists both slow the rate of progression and reduce proteinuria [18-20]. Our data show that this nephro-urological group of patients is no exception.

While VUR patients with abnormal bladders almost invariably present in early childhood, patients with normal bladder function have a bimodal presentation. In one series from New Zealand, 42 patients (36 adults) had ESRF with reflux nephropathy. Many had presented with advanced renal insufficiency, hypertension, and proteinuria, and only 22% of males and 58% of females had a history of UTI. Similarly, in our study, VUR had been proven in 50% of patients with primary reflux nephropathy and these patients (71% female) had almost invariably presented with an UTI in childhood (median age 7.0 years). In contrast, the other half (32% female) presented at a median age of 24 years with advanced disease and persistent reflux was not demonstrated in the 2 patients who underwent a MCU. Similar to the New Zealand experience [5,21,22], nearly all our women presented with hypertension after starting a contraceptive pill or with complications during pregnancy, whereas the men were found to have proteinuria, hypertension or renal insufficiency – usually on routine investigation.

Although reflux nephropathy is frequently viewed as a disease of little girls with recurrent UTIs [7], our data confirms findings of others regarding the late presentation of adults with asymmetric irregular kidneys. In a UK series from Newcastle, only 9% presented under 20 years of age [2], in Australia 22% under 15 years [4], and from Italy 10% under 12 years [6]. Furthermore it is clear from published reviews [2,4-6] and dialysis programmes [5,23], that there is no female preponderance at ESRF.

In 1978 Kincaid-Smith and her colleagues [12] reported that progressive renal failure with primary VUR was very unlikely unless proteinuria was in excess of 1.0 g/day (equivalent to 100 mg protein/mmol creatinine). In a subsequent report, in which 147 such patients were followed for a mean of 6.9 years, renal function deteriorated in 37% and 14% progressed to ESRF. Proteinuria, elevated creatinine and hypertension at presentation were associated with relative risks (RR) of 25, 24 and 4.5 respectively for the development of progressive renal failure [4].

In an Italian study [6], 80 patients were followed for a mean of 5.6 years and retrospectively stratified into those with stable renal function and those with slowly or rapidly progressive renal failure. For those with progressive nephropathy, there was no difference in initial renal function but proteinuria was much greater in the rapidly progressive group. Loss of function was unusual with creatinine ≤ 1.7 mg/dl (150 μmol/l) and inevitable above that concentration [6].

In a UK study from Newcastle, proteinuria and renal insufficiency (plasma creatinine >130 μmol/l) were present from presentation in 21% and 13% of 125 patients respectively, and with time (mean 5.9 years) a further 21% developed proteinuria and 22% renal insufficiency. In all the 16 patients with progressive renal failure, the decline was linear. In a subsequent report, progressive renal failure did not develop in 138 adult patients with normal function at the start (plasma creatinine < 90 μmol/l) [3].

Our data support the established relationship between the risk of progressive nephropathy and renal insufficiency with proteinuria, but suggests a watershed range for renal function as a predictor of outcome. When the eGFR exceeds 40–50 ml/min/1.73 m^2 ^nephropathy rarely progresses, but disease progression is invariable when function is worse. Nakashima et al. similarly reported that an isotopic GFR less than 49 ml/min predicted decline to ESRF [24].

The other determining factor of poor renal outcome is proteinuria, and we find that deterioration can be expected when proteinuria exceeds 50 mg/mmol (0.5 g/d). In a Japanese study, serial biopsy samples from patients with reflux nephropathy confirmed the close association between the degree of renal scarring, the extent of the glomerular pathology, and proteinuria [25,26]. Once extensive glomerular sclerosis was present there was conspicuous glomerular hypertrophy which correlated with increasing proteinuria. This is consistent with our findings that proteinuria increased as renal function declined. The prognostic importance of proteinuria is emphasised by our observation that 6 of the 25 (24%) patients with proteinuria ≥ 200 mg/mmol developed ESRF despite initial eGFRs exceeding 40 ml/min. On the other hand, the survival outcome benefit of ACEI treatment was most conspicuous when patients with proteinuria = 100 mg/mmol were compared (p < 0.00001).

In a 10-year follow up study of 52 children, randomised to medical or surgical management of severe bilateral VUR (grades III-IV) between 1985–1989, progressive renal failure developed in only 4 children (2 from each group) all of whom had GFRs at or below 40 ml/min/1.73 m^2 ^at outset [27].

Despite the few long-term studies of adult patients with primary VUR and reflux nephropathy [2-5], there is almost no renal outcome data on adult patients born with abnormal bladder function (Group 2) [28]. It had been our clinical impression that those with secondary reflux did less well, but although there was a trend for Group 2 patients to do less well this was not statistically significant, although a difference might emerge if large numbers were studied.

Progressive renal damage due to congenital outflow tract obstruction may be averted by urological intervention. This is not, however, always successful and even treatment *in utero *may not prevent progressive renal damage [29] and the development of ESRF. Despite correction of urethral obstruction, 30% of boys with PUV develop ESRF by the age of 15 years and this may be due to continuing bladder dysfunction [28]. Most boys born with abnormal bladders who develop ESRF have posterior urethral valves, but in our series 35% had presented with megacystis/megaureters. Before 1975, this was attributed to bladder neck obstruction and treated by bladder neck surgery but subsequently it has been determined that most of this group are born with gross bilateral VUR. The dilated bladders and ureters are attributed to the constant recycling of refluxed urine, sometimes exacerbated by the nephrogenic polyuria [30] although urodynamic studies often show high voiding pressure suggestive of detrusor/sphincter dyssynergia. The concept that progressive renal failure is often `nephrogenic' in origin, rather than urological, is supported by our data which show that adults with abnormal bladders do not behave significantly differently to those with primary reflux – although the numbers are relatively small. Nevertheless, if renal function deteriorates in a urological patient in the *absence *of proteinuria, then some other cause (such as obstruction) must be sought.

Apart from small numbers, the limitations of this study are the usual ones in observational research with effect estimates that are potentially confounded. We believe, however, that it would not now be possible to design a prospective study in which ACEI therapy was with held from one group. In this study, ACEI therapy improved renal outcome although non-ACEI patients were generally from an earlier period and not necessarily seen in a specialist clinic. The analysis demonstrated a long-term benefit for ACEI both in slowing the rate of loss of function and in reducing proteinuria. However, ACEI therapy appeared to be ineffective if started when the eGFR was already = 30 ml/min.

We have studied a group of patients whose renal pathology is not immunological and whose residual functioning tissue is not homogenously distributed. The pathophysiology is similar to experimental sub-total nephrectomy which results in proteinuria, progressive glomerulosclerosis and renal failure [13,31]. Like the experimental models, we have found that there is a watershed level of GFR above which ESRF is unlikely and below which it is invariable, and that the most important harbinger of poor outcome is proteinuria. Why, however, one patient with a GFR of 45 ml/min should have no proteinuria and do well, while another with a GFR of 50 and 2 g/day of proteinuria does badly, remains to be determined. Long term studies [27] and follow up observations on outcome [23] in patients with reflux nephropathy confirm no benefit from anti-reflux surgery. This is consistent with our current view that progressive nephropathy is nephrogenic rather than urological in origin.

## Conclusions

Patients with a GFR of <60 ml/min need careful follow up and we would recommend that anyone with increasing proteinuria, or proteinuria >50 mg/mmol (0.5 g/d) is started on an angiotensin antagonist to reduce proteinuria and slow the rate of progression of the renal failure.

## Competing interests

GHN has been a consultant and lectured for MSD regarding both ACE inhibitors and angiotensin II receptor blockers. G Thomson, D Nitsch, RG Woolfson, JO Connolly, and CRJ Woodhouse have no conflict of interest.

## Authors' contributions

GN was responsible for the study design, management of patients, and writing of the report. GT collected clinical data from medical records; DN contributed to analyses and writing of the report; RW and JC contributed to patient management and writing of the report. CW was responsible for the continuing urological supervision of the patients and writing of the report. All authors read and approved the final manuscript.

## Pre-publication history

The pre-publication history for this paper can be accessed here:


